# Identification of Lipid Species Signatures in FOLFOXIRI-Resistant Colorectal Cancer Cells

**DOI:** 10.3390/ijms26031169

**Published:** 2025-01-29

**Authors:** George M. Ramzy, Isabel Meister, Serge Rudaz, Julien Boccard, Patrycja Nowak-Sliwinska

**Affiliations:** 1 Molecular Pharmacology Group, School of Pharmaceutical Sciences, University of Geneva, 1211 Geneva, Switzerland; george.ramzy@unige.ch; 2 Institute of Pharmaceutical Sciences of Western Switzerland, University of Geneva, 1211 Geneva, Switzerland; isabel.meister@unige.ch (I.M.); serge.rudaz@unige.ch (S.R.); 3 Translational Research Center in Oncohaematology, 1211 Geneva, Switzerland; 4 Department of Cell Physiology and Metabolism, Faculty of Medicine, University of Geneva, 1211 Geneva, Switzerland; 5 Biomedical and Metabolomics Analysis Group, School of Pharmaceutical Sciences, University of Geneva, 1211 Geneva, Switzerland; 6 Swiss Centre for Applied Human Toxicology (SCAHT), 4055 Basel, Switzerland

**Keywords:** colorectal carcinoma, drug combination, FOLFOXIRI, lipidomic, mass spectrometry, acquired drug resistance, sphingolipids, triglycerides

## Abstract

Chronic drug treatment can alter the lipidome of cancer cells, potentially leading to significant biological changes, such as drug resistance or increased tumor aggressiveness. This study examines the lipidome profiles of four human colorectal cancer (CRC) cell lines, comparing treatment-naïve cells with the same cells after chronic exposure to a clinically used combination therapy (FOLFOXIRI: folinic acid, 5-fluorouracil, oxaliplatin, and irinotecan). Lipidomic profiling was obtained with untargeted liquid chromatography coupled with high-resolution mass spectrometry (LC-HRMS). For data deconvolution and to interpret the multifactorial dataset generated, Analysis of Variance Multiblock Orthogonal Partial Least Squares (AMOPLS) was used. Our results indicate specific shifts in triglycerides (TGs), sphingolipids, and phospholipids in CRC cells resistant to FOLFOXIRI. The overall shift in TGs, phosphatidylcholine, and cholesteryl ester species was notably linked to FOLFOXIRI resistance (-R) in SW620 cells, whereas an increased abundance of phospholipids, mainly hexosylceramide and sphingomyelin, was present in the signatures of HCT116-R, LS174T-R, and DLD1-R cells. These altered lipid species may serve as potential prognostic markers in CRC following chemotherapy. Furthermore, lipid-targeting therapies aimed at reprogramming the lipid profiles of drug-resistant cells could play a crucial role in restoring drug sensitivity and improving patient survival.

## 1. Introduction

Colorectal cancer (CRC) ranks globally as the third most commonly diagnosed and second deadliest cancer worldwide, with approximately 1.9 million new cases in 2020 projected to increase to 3.2 million new cases and 1.6 million deaths in 2040 [1]. The management of CRC depends on various factors, including the stage of the disease, the molecular subtype of the tumor, and the patient’s comorbidities [2]. The main treatment modality for localized CRC consists of surgical resection of the primary tumor, often followed by adjuvant therapy [3]. The backbone of CRC treatment is the administration of a combination of chemotherapy consisting of folinic acid (FOL), 5-fluorouracil (F), oxaliplatin (OX), and irinotecan (IRI) [4]. Depending on the stage of the tumor and the state of the patient, these chemotherapeutics can be combined (FOLFOX, FOLFIRI, or FOLFOXIRI) or given as monotherapies in combination with targeted treatments [2,5]. Despite being the standard of care, this treatment modality comes with a large spectrum of adverse effects that greatly impact the patient’s quality of life, and efficacy is compromised over time as most patients develop acquired drug resistance. The CRC patient population is relatively heterogenous, with various mutation signatures or consensus molecular subtypes [6]. This highlights the necessity of investigating multiple samples to represent the intra-patient heterogeneity, achieved in laboratory settings by using various immortalized cell lines isolated from patients.

**Figure 1:**
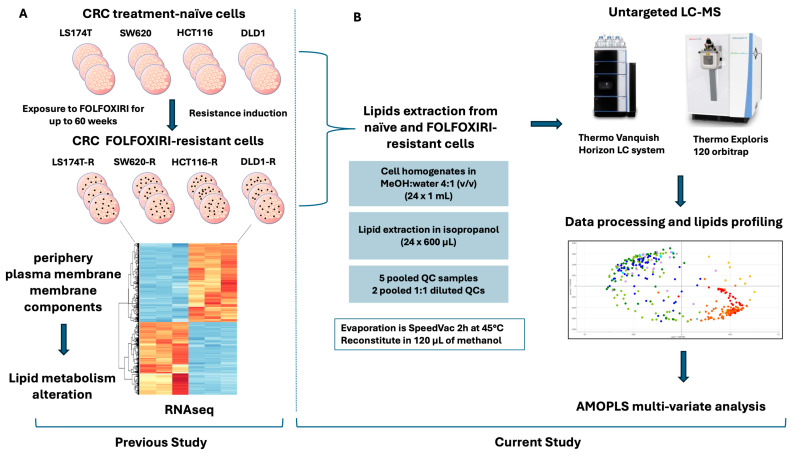
Study design. Past study revealing lipid metabolism alterations [7] (**A**). Current study design (**B**).

In our previous work, we established a CRC in vitro model to mimic the clinical state of patients diagnosed with late-stage CRC who underwent a long-term treatment with FOLFOXIRI [7]. By chronically exposing human CRC cells to the chemotherapy combination [8], acquired FOLFOXIRI resistance was obtained over time. Next, our results obtained in bulk RNA sequencing in FOLFOXIRI-resistant cells confirmed that the acquired chemoresistance was linked to a dysregulation of genes implicated in the periphery, plasma membrane, and membrane components; this underscores a strong alteration in lipid metabolism, see Figure 1A. This was a starting point for the current study.

Lipids are implicated in crucial cellular functions such as membrane formation and intracellular compartmentalization, as well as signaling processes and energy depots [9]. Cellular lipids are constantly changing in response to the physiological, pathological, and environmental conditions [10]. We were therefore interested in investigating these lipid alterations in the context of CRC and FOLFOXIRI resistance in greater detail using untargeted lipidomic profiling. The study of the lipidome, which aims to provide the most comprehensive lipid profile within a cell, has gained a lot of attention in recent years, fueled by analytical and computational advances [11]. Recent studies have highlighted strong associations between changes in the lipidome and various tumor types. Pakiet et al. elegantly discussed the multiple alterations in lipids in the context of CRC, with a focus on fatty acids as a potential diagnostic biomarker [12]. Multiple studies have presented the changes in lipid metabolism upon acquired resistance to a given therapy [13,14]. The implications of lipid changes in the mechanisms of resistance to conventional chemotherapy have been also investigated in the context of CRC. However, those studies only included resistance to one [15,16,17,18] or two drugs [19] from the chemotherapy combinations. Investigations performed on different patient cohorts have underlined that the main difference between healthy colon and cancerous colon tissues lies in a higher concentration of sphingolipids, specifically sphingomyelins and triglycerides in colon cancer tissue [20,21].

In this study, we present a comparison of the lipid signatures of a panel of CRC treatment-naïve and FOLFOXIRI-resistant cells using untargeted liquid chromatography coupled with high-resolution mass spectrometry (LC-HRMS)-based profiling. The dataset generated was analyzed thanks to a multivariate deconvolution approach, namely Analysis of variance-Multiblock Orthogonal Partial Least Squares (AMOPLS). This is a linear method specifically developed to separate the different effects when structured experimental factors are used, while conventional multivariate data analysis methods such as Principal Component Analysis (PCA) or Partial Least-Squares Discriminant Analysis (PLS-DA) fail in this context. It should be noted that the aim was to explain specific variations related to each effect rather than predict or classify observations. It is indeed crucial to assess biochemical and functional relationships reliably [22], thus AMOPLS was used to offer a deeper understanding of lipidome alterations associated with the acquisition of resistance to FOLFOXIRI (Figure 1B).

## 2. Results

### 2.1. Lipid Signature Shift in CRC FOLFOXIRI-Resistant Cells

We used four well-characterized primary human colon carcinoma cell lines, see Table 1. These four cell lines harbor different mutational backgrounds covering diverse CRC patient profiles, making them valuable models for studying the variability in CRC responses to treatment. These cells were exposed chronically to FOLFOXIRI, a first-line standard of care for late-stage CRC patients, for up to 60 weeks, and resistant cell clones were established as described previously [7,8]. This combination, as opposed to FOLFOX or FOLFIRI, contains the full tri-therapy given for patients with the most advanced stage of the disease.

**Figure 2:**
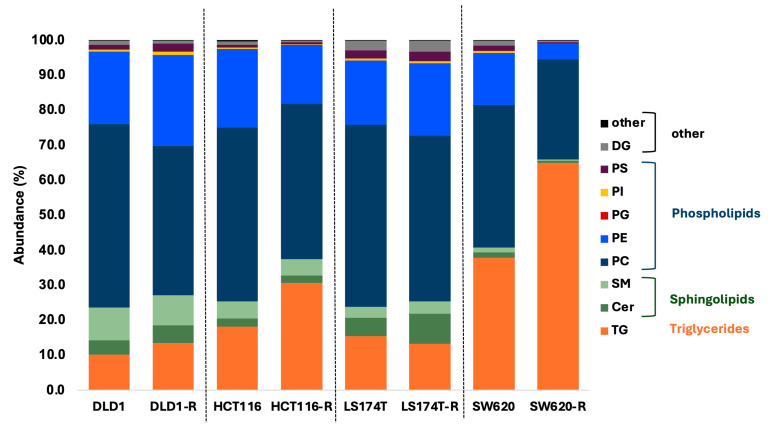
Normalized distributions of the main lipid sub-classes in FOLFOXIRI-naïve and FOLFOXIRI-resistant (-R) clones. DG = diacylglycerol; PS = phosphatidylserine; PI = phosphatidylinositol; PG = phosphoglycerol; PE = phosphatidylethanolamine; PC = phosphatidylcholines; SM = sphingomyelin; Cer = ceramides; TGs = triglycerides.

The global lipidome of the selected FOLFOXIRI-naïve cells, involving 1737 MS2-matched lipids, showed specific lipid signatures (Figure 2). HCT116 and LS174T cells showed similar lipid compositions, with 73% phospholipids and 7–8% total sphingolipids (with a higher content of ceramides in LS174T cells (TNM 2), which is in line with previous studies [28]), and 15–18% TGs (Supplementary Table S1). DLD1 cells exhibited the highest relative amount of sphingolipids among all four cell lines (14%), while the SW620 cell line presented the highest proportion of triglycerides (TGs) (38% compared to 10–15% in the three other cell lines). Globally, the common lipid phenotype among all cell lines is very weak: we can see an overall similar composition between DLD1, HCT116, and LS174T cells, contrasting with the SW620 lipidome, which is characterized by lower relative amounts of polar lipids and a higher triglyceride content.

We next assessed the lipid signature shift associated with induced FOLFOXIRI resistance in CRC cells compared to their FOLFOXIRI-naïve counterparts. Our results showed that lipid reprogramming is cell line specific and does not necessarily show strong common patterns in CRC FOLFOXIRI-resistant cells (-R). DLD1-R, HCT116-R, and SW620-R cells presented increases in TG abundance of 3%, 13%, and 27%, respectively (Figure 2 and Supplementary Table S1), as well as a rise in all ether lipids (ether-TGs, ether-phosphatidylcholine (ether-PC), and ether-phosphatidylethanolamine (ether-PE)). Interestingly, both trends were reversed in LS174T-R cells, which showed decreases in all TGs and all ether lipids. In addition, resistance to FOLFOXIRI in LS174T-R cells was associated with a slight increase of 4% in the relative amount of sphingolipids, especially ceramides.

### 2.2. Experimental Design Investigation Using AMOPLS

**Figure 3:**
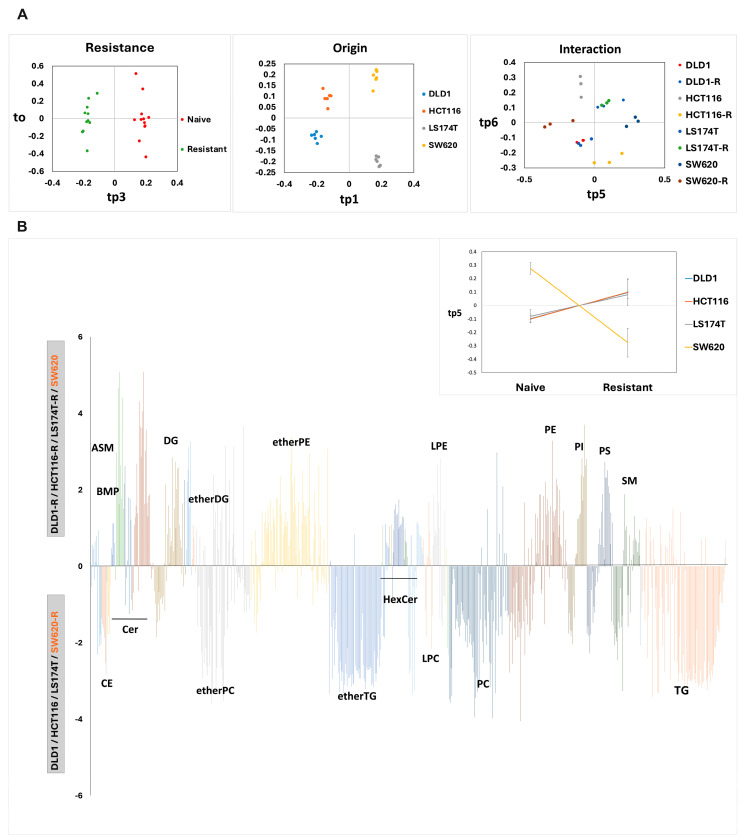
AMOPLS analysis. Multifactorial analysis computed including the FOLFOXIRI resistance main effect on 2 levels: FOLFOXIRI naïve or resistant (left panel, *X*-axis corresponding to predictive component 3 (Tp3) and *Y*-axis to tpo); cell origin main effect on 4 levels: DLD1, HCT116, LS174T, and SW620 cells (middle panel, *X*-axis corresponding to tp1 and *Y*-axis to tp2); and origin × resistance interaction effect (4 × 2 levels) (right panel, *X*-axis corresponding to tp5 and *Y*-axis to tp6) (**A**) Loading plot of AMOPLS predictive component linked to the origin × resistance main effect (**B**).

AMOPLS was then calculated, including a fixed FOLFOXIRI-resistance factor (two levels: naïve, resistant) and a fixed cell origin factor (four levels: DLD1, HCT116, LS174T, and SW620 cells), leading to a model accounting for their main effects and their potential interaction (origin × resistance), as previously described [29,30], to assess the corresponding shifts in the lipidome (Supplementary Table S2). The AMOPLS model separates the proportion of each effect within the dataset: resistance = 7.8%, origin of the cells = 58.4%, interaction (origin × resistance = 18.1% and residuals = 15.7% (unexplained part of the observed variability). A single orthogonal component was found to be optimal according to random permutations (with R^2^ = 0.97). All the main and interaction effects were observed to be highly significant (*p* < 0.0001). AMOPLS also provides a decomposition of the trends associated with each effect, summarized using predictive components, as a function of the amplitude of the measured differences in the data. We first investigated the common lipid shift between all four cell lines due to the acquired FOLFOXIRI resistance (main effect) (Figure 3A, left panel).

This common trend, recognized as a resistance phenotype, was shown to be associated with higher levels of TGs, etherTG, CE, ceramides (Cers), hexosylceramides (HexCers), and lysophosphatidylcholine (LPC) in the FOLFOXIRI-resistant cells compared to their FOLFOXIRI-naïve counterparts (Supplementary Figure S1). FOLFOXIRI resistance was also associated with a decrease in acid sphingomyelinase (ASM), bis(monoacylglycero) phosphate (BMP), diglycerides (DG, etherDG, etherMGDG), PE, LPE, phosphatidylinositol (PI), and phosphatidylserine (PS). Interestingly, SM, PC, and etherPC did not show a strong shift representative of entire classes but rather shifts linked to individual species. In etherPC, we were able to observe a trend for a decrease in longer-chain species and an increase in shorter-chain species for the FOLFOXIRI-resistant phenotype. Furthermore, we observed a clear distinction between all four cell lines linked to the origin of the cells (main effect). The first predictive component summarized 39.7% of the total variance, separating SW620 and LS174T from HCT116 and DLD1, while the second held 14.9% of the total variance (Figure 3A, middle panel), revealing lipid signatures characterizing the different cell origins (Supplementary Figure S4). HCT116 cells were associated with higher levels of PC and BMP; SW620 cells with TG and etherTG; DLD1 with SM and most Cers and HexCers; LS174T with etherPC and HexCer_HS. Finally, the AMOPLS components summarizing lipid shifts due to resistance in a cell-line-specific manner were then considered to assess the interaction effect (origin × resistance) (Figure 3A, right panel). We can easily distinguish the two lipid phenotypes, previously defined as seen in Figure 2, with SW620 cells representing a particular shift in lipids compared to the other cell lines. The most salient shift in lipids was associated with SW620 cells, revealing an alteration mainly linked to increased TG and etherTG levels, while subspecies of ceramides (Cer_NS, Cer_HDS, Cer_HD) were found to be decreased in this cell line compared to the other three (Figure 3B).

### 2.3. Cell-Line-Specific Lipid Shift

**Figure 4:**
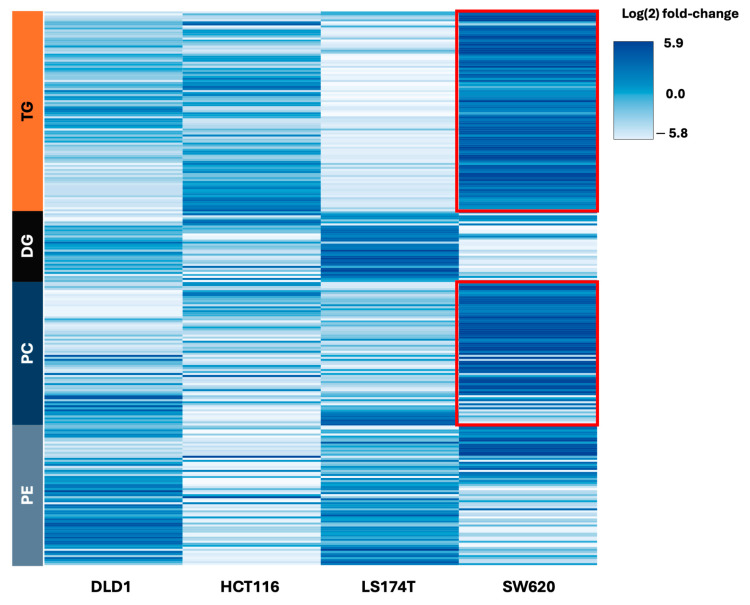
Heatmap of the log (2) fold changes in the abundances of selected lipid families in FOLFOXIRI-resistant/-naive cells. Red boxes highlight the signature shifts between SW620 vs. DLD1,HCT116 and LS174T, upon exposure to FOLFOXIRI.

The variance analysis in the AMOPLS analysis highlighted the main common variation in lipids between all four cell lines (Supplementary Figure S3). The contribution was, however, low compared to the effects of the cell line and resistance combined (7.8% for the resistance vs. 18.1% for the interaction), stressing the interest in investigating cell-line-specific resistance patterns. Score plots identified SW620 cells as behaving differently from the other cell lines (DLD1, HCT116, and LS174T) in response to chronic exposure to FOLFOXIRI). To investigate this effect in more detail, we focused on a selection of lipids (Figure 3B) that were selected based on their analytical performance, including chromatographic peak shape, repeatability, and signal-to-noise ratio. The SW620-specific resistance pattern can be summarized as having a much higher increase in TGs compared to the other cell lines, as shown in Figure 2 and further validated with a large fold-change increase (log(2) fold change > 5), as seen in the heatmap of Figure 4. Furthermore, the patterns of PCs were not well defined in the common FOLFOXIRI-based resistance profile, but we can observe that SW620 cells had a marked increase in several of these lipids in the resistance phenotype, while in the other cells they appear to decrease, as confirmed in Figure 4.

**Figure 5:**
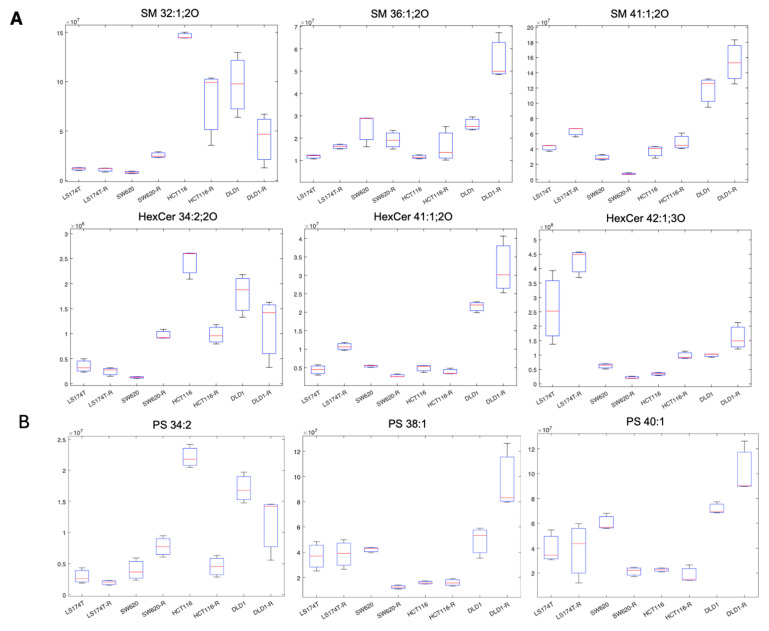
Boxplots of representative SM and HexCer species (**A**) and PS (**B**), showing different behavior in respect to chain length for SW620 cells compared to the other cell lines.

TGs and PCs clearly show a global marked increase in SW620-R cells compared to the other cell lines, suggesting a specific lipid signature shift linked to FOLFOXIRI resistance. This distinct behavior was not observed in other neutral lipids (DGs) or other phospholipids (PEs). Cers, which tended to increase HCT116-R, LS174T-R, and DLD1-R cells, displayed decreased abundances in SW620-R cells. Interestingly, SMs and Hex_Cer showed different behaviors according to their acyl chain length: 18 carbon or longer chains (i.e., SM 36:1;2O; SM 41:1;2O, and HexCer 42:1;3O) tended to decrease in SW620-R cells and increase in the DLD1-R, HCT116-R, and LS174T-R cells, while shorter-chain lipids (i.e., SM 32:1;2O and HexCer 34:2;2O) adopted the opposite behavior, see Figure 5A. Furthermore, PS also seemed to show differences in shifts according to its chain length. In this case, a reduction in the phospholipids in the resistant phenotype is amplified in longer-chain PS and dimmed in short-chain PS for SW620 cells (i.e., PS 34:2 vs. PS 40:1) compared to the other cell lines (Figure 5B). Globally, these results are in line with our normalized distribution of the main lipid sub-classes presented in Figure 2 and further underline the two distinct lipid phenotypes observed.

## 3. Discussion

Changes in lipid metabolism originate from both an intrinsic cell-line-specific and treatment-acquired manner, resulting in high heterogeneity and variability. The lipid metabolism changes in cells with induced resistance could be related to two different processes. The first consists of an adaptive process translated through a metabolic shift in the cancer cells in response to treatment-induced cellular stress, such as a lack of nutrients or the presence of reactive oxygen species. The second consists of the selection of pre-existing cellular sub-populations with aberrant lipid metabolism [14]. Our previous findings showcased that the FOLFOXIRI-resistant cells acquired resistance towards the full tri-therapy and not exclusively to single agents [7]. This resistance was shown to be mainly linked to the altered expression of genes implicated in lipid metabolism. In this study, we mainly looked for the adaptive process after chronic FOLFOXIRI treatment of cells, as previously described^7^, in terms of lipid shifts in four different human CRC cell lines. We first compared the lipidome profile of the FOLFOXIRI-resistant cells with matched FOLFOXIRI-naïve cells (Figure 2). We observed that after chronic treatment with FOLFOXIRI, SW620 cells underwent the most visible lipids shift, mainly in TGs and phospholipid families. Globally, these results allowed us to define two distinct lipid phenotypes observed within the framework of the study: SW620 vs. HCT116, DLD1, and LS174T resistant phenotypes (SW620-R vs. HCT116-R, DLD1-R, and LS174T-R). To further investigate the shifts in lipid signature, we used an AMOPLS model. This method has the advantage of combining variance decomposition using standard ANOVA and the OPLS framework, separating predictive from orthogonal variations using specific component(s). AMOPLS was reported as an efficient tool that provided clearer separation of factor levels, thus offering easier interpretation of the variation trends [30]. The lipidomic data analysis using predictive components linked to common resistance in AMOPLS showcased a rather weak common pattern between all four cell lines in lipid shifts, with mainly an increase in TGs (TG and etherTG), Cer, LPC, CE, and short-chain etherPC species in FOLFOXIRI-resistant cells (Supplementary Figure S3). In the next step, we analyzed the shifts in lipids specific to each of the four cell lines. The predictive component linked to origin x resistance showed that the overall shifts in TG, etherTG, etherPC, and CE species were notably linked to the FOLFOXIRI resistance in SW620 cells (Figure 3B, Figure 4). TGs play an important role as transporters of fatty acids, as well as serving as an energy source to the cells by metabolically breaking down into fatty acids and glycerol, after which both can serve as substrates for energy-producing and metabolic pathways [31]. Such an increase in TGs can be linked to an accumulation of lipid droplets (LDs) in the cells, which has been shown to be a hallmark of cancer and correlated with CRC relapse [32]. Cotte et al. showcased an increased synthesis of LDs via the upregulation of LPCAT2 (enzyme in charge of PC synthesis). The accumulation of LDs in SW620 cells was previously linked to chemotherapy resistance, mainly to 5-FU and oxaliplatin, in vitro and in vivo by blocking chemotherapy-related ER stress. However, the intensity of this phenomenon was cell line specific [19], which is in line with our findings, where the increase in TGs was observed in all cells upon FOLFOXIRI exposure but with a particularly marked intensity in SW620-R. In recent findings, Larson et al. showcased that untargeted proteomic analysis on HCT116 oxaliplatin-resistant cells confirmed the upregulation of LD-related proteins in association with an increased abundance of TG. Interestingly, this increase was not related exclusively to the resistant phenotype but was also found in the oxaliplatin-sensitive cells upon punctual treatment with oxaliplatin [33]. Furthermore, the shift in TG lipids in the FOLFOXIRI-resistant cells, which we linked to a potential accumulation of LDs, can result in an increased storage of CE in the resistant cells, which further confirms our findings (Figure 3B). An excessive abundance of CE in cancer cells has been shown to promote aggressiveness and metastasis in multiple cancer types, including CRC [34,35], and was recently highlighted in CRC patient samples exposed to clinical FOLFOX (5-FU, folinic acid, and oxaliplatin) [36]. In addition, we observed an abundance of ether lipids in the FOLFOXIRI-resistant cells in a species-dependent manner: etherDG and etherPE in DLD1-R, HCT116-R, and LS178T-R and etherTG and etherPC in SW620-R, see Figure 3. This highlights an adaptive cell-line-dependent process towards FOLFOXIRI. Ether lipids (etherTG, etherPC, and etherPE) have been shown to be highly abundant in cancer cells, and increases in their levels are often linked to a more aggressive phenotype in multiple cancer types [37]. This was also confirmed in our previous study, where the same CRC FOLFOXIRI-resistant cells were used and had a more “diffusing” phenotype, being less round and more elongated, and acquired a more aggressive morphology [7]. Therefore, one may consider this technology to study one aspect of disease progression, providing that a sample from a patient could be provided during disease development. Furthermore, the predictive component linked to origin x resistance showed that the overall shift in TGs and PCs was less marked in the second lipid phenotype linked to the HCT116-R, DLD1-R, and LS178T-R cells. However, we have also detected a common increase in phospholipids, specifically hexosylceramides (HexCer) and SMs, in these cells (Figure 3B, Figure 5A). Sphingolipids are major components of the cell membrane lipidome and are therefore implicated in structural functionality during cell proliferation and/or apoptosis [38]. Dysregulation in the metabolism of this family of lipids, specifically ceramides, has been shown to play a prominent role in chemotherapy resistance in different types of cancer, including breast and melanoma [39], and specifically to 5-FU in CRC cells [16],. In addition, dysregulation in the metabolism of sphingolipids was shown to sensitize tumor-resistant cells to chemotherapy in prostate adenocarcinoma [40,41]. Ceramides have been shown to induce apoptosis in cancer cells [42]; therefore, the reduction or conversion of ceramides has been considered as a cancer resistance mechanism in multiple cancer types like leukemia [43,44] and breast cancer [45]. HexCers are obtained through the enzymatic addition of a sugar molecule to the backbone ceramide molecule, resulting in a more complex sphingolipid. The conversion of ceramides through glycosylation into HexCers was previously linked to the resistance to 5-FU and cisplatin in breast cancer cells [46]. This could explain the resistant phenotype observed in HCT116-R, DLD1-R, and LS174T-R cells linked to an increased abundance of HexCers. In addition, in CRC an altered metabolism of sphingolipids has been linked to a more aggressive phenotype via the promotion of metastasis through epithelial–mesenchymal transition [47,48].

This study had some limitations that are worth noting. We used an example of only four CRC cell lines to represent patient heterogeneity. The cell line characteristics presented in Table 1 are not exhaustive. It remains to be demonstrated which of these background factors might be influencing our lipidome results. Another point that needs further investigation is which specific drug or combination of drugs within the FOLFOXIRI tri-therapy is responsible for the observed lipidome changes, as it might very well be that not all drugs contribute equally to our observed results. This type of deconvolution is currently being investigated in our laboratory. Although laborious due to the nature of the data analysis used in this study, the number of samples used could be increased to better represent the patient population. Moreover, our analysis was performed in cancer (immortalized) cells. This was a carefully considered decision, as these cells are well-characterized and stable. We are aware that this simplified model does not address cellular crosstalk or the tumor microenvironment present in tumor tissue. Finally, although AMOPLS is suitable for handling datasets involving multiple experimental factors, interpreting the model can be challenging. Understanding how different factors interact and contribute to the results requires careful consideration of the experimental design and expertise in assessing the observed differences between groups. In addition, AMOPLS assumes linear relationships between variables. If the true relationships are nonlinear, the method may not capture the underlying patterns effectively.

## 4. Materials and Methods

### 4.1. Cell Lines and Culture Conditions

Human CRC cells (Table 1) were purchased from ATCC or Public Health England, while the resistant counterpart from each was generated as previously described^7^. HCT116 (RRID: CVCL_0291), HCT116-R (RRID: CVCL_C4RX), LS174T (RRID: CVCL_1384), LS174T-R (RRID: CVCL_C4RY), SW620 (RRID: CVCL_0547), and SW620-R (RRID: CVCL_C4RZ) cells were cultured and maintained in DMEM Glutamax medium (31966-021, Gibco, Gaithersburg, MD, USA), while DLD1 (RRID: CVCL_0248) and DLD1-R (RRID: CVCL_C4RW) cells were cultured and maintained in RPMI-1640 Glutamax medium (1870-010, Gibco). Both culture media were supplemented with 10% fetal bovine serum (S1810-500, Biowest, Nuaillé, France) and 1% penicillin/streptomycin (4-01F00-H, Bioconcept, Basel, Switzerland). Cells were kept in a humidified atmosphere at 37 °C with 5% CO_2_ (Binder). All cells were tested for mycoplasma (MycoAlert cat. LT07-218) presence before all experiments.

HCT116, DLD1, LS174T, and SW620 cells underwent the cell line typing service provided by Microsynth AG (Balgach, Switzerland). For this, 1.5 million cells from each cell line were collected, washed with PBS, and then the cell pellet suspended in 70% ethanol.

### 4.2. Sample Preparation for Lipidomic Analysis

Cells (2D cell culture system) were prepared for lipidomic profiling by first washing 2 × with 4 mL 150 mM ammonium acetate, then metabolism was quenched by adding 1 mL extraction solution (cold MeOH + 0.16 µM LPC 18:1-d7). The cells were then scraped and the suspension transferred to 1.5 mL Eppendorf tubes and stored at −80 °C until being assayed. For the lipid extraction, samples were thawed at 4 °C for 2 h and centrifuged for 15 min at 14,000× *g* and 4 °C. A total of 600 µL of the supernatant was collected into a new 1.5 mL Eppendorf and evaporated in a SpeedVac for 1 h using a low-temperature setting. For the constituting the pooled QC, an aliquot of 310 µL of the supernatant of each sample was pooled and aliquoted in 5 × 600 µL aliquots. Samples and QCs were evaporated to dryness in a SpeedVac (Thermo Fisher, Waltham, MA, USA) for 1 h using a low-temperature setting. On the day of analysis, samples were reconstituted in 120 µL of methanol, vortexed for 20 s, and shaken for 1 h at 4 °C and 900 rpm in a Thermomix (Eppendorf, Hamburg, Germany). To avoid particulates in the LCMS system, samples were centrifuged for 15 min at 12,000× *g* and 4 °C. A total of 110 µL of supernatant was transferred to LC-MS vials. Blank samples consisted of the resuspension solvent only. The system suitability test (SST) sample consisted of the UltimateSplash^®^ mix from Avanti Lipids (Alabaster, United States) containing 72 deuterated lipid standards (1:50 *v/v* diluted with methanol).

### 4.3. LC-HRMS Lipidomic Measurements

Untargeted lipidomic profiles were measured on a Vanquish Horizon LC system (Thermo Fisher) with a BEH C18 Premier LC column (2.1 × 100 mm, Waters, Milford, MA, USA) connected to a high-resolution Exploris 120 orbitrap mass spectrometer (Thermo Fisher). The mobile phase A was 10 mM ammonium acetate in acetonitrile/water 6:4 (*v*/*v*) and mobile phase B of 10 mM ammonium acetate in isopropanol/acetonitrile 9:1 (*v*/*v*). The elution gradient ran with a flow rate of 0.4 mL/min from 20% to 60% B in 3 min, then to 85% in 7 min and to 97% in 5 min, followed by re-equilibration at the initial conditions for 4.5 column volumes. The LC flow was diverted to waste at 15 min. The column oven temperature was set at 50 °C and the injection volume was 3 µL. The mass spectrometer was operating in positive mode (3.4 kV), with internal mass calibration enabled at the start of each run. The source settings were 55 AU for the sheath gas flow, 13 AU and 350 °C for the auxiliary gas, 2 AU for the sweep gas, and the ion transfer tube temperature was set at 340 °C. S-lens RF was set at 70. Each sample was acquired in centroid mode with an alternating full scan at a 60,000 resolution on a mass range of 200–1200 *m*/*z* and top 4 DDA scans at 15,000 resolution with normalized collision energy of 20 eV. The AGC target was set at 100%, with an accumulation time of 80 ms. MS2 scans were triggered with an intensity threshold of 20,000, an apex window of 45%, and a dynamic exclusion of 2 s. A custom exclusion list based on a blank sample was used to avoid fragmenting contaminants.

### 4.4. Data Pre-Treatment

Data were converted to the mzML format using MSConvert software [49] and pre-processed in MS-DIAL software [50]. Peak detection was performed with 0.01 Da mass tolerance, a minimum peak height of 200, a mass slice of 0.1 Da, and smoothing using a linear weighted moving average with smoothing of 3 scans and minimum peak width of 5 scans. Deconvolution of MS2 data was performed with a sigma value of 0.5 and an abundance cutoff of 100. Identification relied on an embedded lipid library with a mass tolerance of 0.01 Da (MS1and MS2), and alignment was performed on the second QC sample with a retention time tolerance of 0.15 min and MS1 tolerance of 0.01 Da. A total of 1737 MS2-matched lipids were automatically annotated with the MS-DIAL internal library (see Supplementary Table S3). Lipid annotations were then curated for an accurate mass < 5 ppm, redundant signals from adducts, and expected elution patterns, resulting in 1309 lipids. Lipids with a missingness rate > 75% across the samples were excluded (missingness threshold = 5 × the signal in blank), as well as those presenting mean intensities in QCs < 1 × 10^5^, CVs in QCs > 40%, and interquartile ranges = 0 (see Supplementary Figure S3A,B). After filtering, 747 lipids were retained in the final dataset. We applied PQN correction based on median QC intensities to account for variations in terms of biomass between the samples (see Supplementary Information S1, Supplementary Figure S4A,B for details).

### 4.5. Data Analysis

AMOPLS was computed after unit variance scaling under the MATLAB^®^ 8 environment (The MathWorks, Natick, MA, USA). A series of 10^3^ random permutations were performed to validate the model and assess the statistical significance of the effects.

## 5. Conclusions

In summary, we report shifts in the lipidomic signature associated with chronic treatment of CRC cells with FOLFOXIRI. Exploring lipidomic changes in these cells may provide valuable insights into the pathogenesis of CRC and inform potential treatment development. The comparisons between FOLFOXIRI-resistant and -naïve cells, as well as between SW620-R and the other cell lines presented in this study, highlight cell-dependent lipid alterations associated with both chronic treatment and disease progression. Although targeting specific lipid shifts remains experimental, continued research in this area could increase the cohort of samples analyzed and enhance our understanding of correlations between altered lipids and CRC progression.

## Figures and Tables

**Table 1:** CRC cell lines used in the study and their molecular characterization [23,24,25,26,27].

Cell Line	Patient	Metastatic Potential	TNM	Molecular Subtype	Genomic (in) Stability	Mutations/ Deregulations
LS174T	Female	low	2	CMS3	MSI	KRAS, PIK3CA, BRAF
SW620	Male	high	3	CMS4	MSS, CIN	APC, KRAS, TP53
DLD1	Male	high	3	CMS1	MSI, CIMP	APC, KRAS, PIK3CA, TP53
HCT116	Male	low	1	CMS4	MSI, CIMP	KRAS, PIK3CA

CMS: consensus molecular subtype; TNM: tumor-node-metastasis staging; MSI: microsatellite instability; MSS: microsatellite stability; CIN: chromosomal instability, CIMP: CpG island methylator phenotype.

## Data Availability

Zenodo DOI:10.5281/zenodo.14042562.

## Supplementary Materials

The following supporting information can be downloaded at: https://www.mdpi.com/article/10.3390/ijms26031169/s1.

## Abbreviations

The following abbreviations are used in this manuscript.

AMOPLS	Analysis of Variance Multiblock Orthogonal Partial Least Squares
ASM	acid sphingomyelinase
BMP	bis(monoacylglycero) phosphate
CE	cholesteryl ester
Cers	ceramides
CIMP	CpG island methylator phenotype
CIN	chromosomal instability
CMS	consensus molecular subtype
DG	diglyceride
HexCer	hexosylceramides
LPC	lysophosphatidylcholine
LPE	lysophosphatidylethanolamine
MGDG	monogalactosylDG
MSI	microsatellite instability
MSS	microsatellite stability
PC	phosphatidylcholine
PE	phosphatidylethanolamine
PG	phosphoglycerol
PI	phosphatidylinositol
PS	phosphatidylserine
SM	sphingomyelin
TG	triglycerides
TNM	tumor-node-metastasis staging
