# Supplementary material for: Identification of Lipid Species Signatures in FOLFOXIRI-Resistant Colorectal Cancer Cells
Source: Int J Mol Sci. 2025 Jan 29;26(3):1169. doi: 10.3390/ijms26031169 (PMC11818583; doi:10.3390/ijms26031169)
Supplement: Supplementary file 1 [file ijms-26-01169-s001.zip › ijms-3422925-supplementary.pdf]

Supplementary material

Identification of lipid species signature in FOLFOXIRI-resistant colorectal cancer cells

George M. Ramzy[1,2,3,4], Isabel Meister[2,5,6], Serge Rudaz[2,5,6], Julien Boccard[2,5,6*], Patrycja Nowak-Sliwinska[1,2,3,6*]

*Corresponding authors:

Julien Boccard, 1 Rue Michel-Servet, 1211 Geneva 4, Switzerland,

Tel: +41 22 379 6477, e-mail: julien.boccard@unige.ch

Patrycja Nowak-Sliwinska, 1 Rue Michel-Servet, 1211 Geneva 4, Switzerland,

Tel: +41 22 379 3352, e-mail: Patrycja.Nowak-Sliwinska@unige.ch

Supplementary material

Supplementary information

Supplementary tables S1-3

Supplementary figures S1-4

Supplementary information S1: Samples quality control and peak acquisition

Samples were delivered in two batches (batch 1: SW/SWR and LS/LSR; batch 2: HCT/HCTR and DLD/DLDR). QC samples were constituted at the batch level. To ensure analytical consistency, the two batches were measured sequentially on the same analytical acquisition with respective pooled QC conditioning (see **Supplementary Table S3**). Data quality was assessed using coefficient of variation (CVs) across the QCs and Principal Component Analysis (PCA). The CVs of the technical internal standard LPC 18:1-d7 were 7.5 % and 6.0% for SW/LS and HCT/DLD batches, respectively. The average CV of all lipids assayed was 13.5 % for the SW/LS batch and 7.2% for the HCT/DLD batch. After data filtering and curation, 747 lipids were retained in the final dataset, presenting average CVs of 8.5% and 5.5% in SW/LS and HCT/DLD batches, respectively. The filtering process did not alter the overall data structure, as observed with PCA score plots (**Supplementary Figure S3**). MS drift across both batches was negligeable (no systematic drift was observed across the analytical run) and was therefore not corrected, following the principle of parcimony. To correct the batch-to-batch effect and analyte concentration variation across samples, the PQN algorithm was applied on the whole analytical sequence. The SW/LS QCs were used as reference for PQN, as this batch was lower in terms of MS signal. PCA score plots of before vs. after PQN correction display an excellent clustering of QCs with the 2-fold diluted QCs, showing that the concentration effect was efficiently corrected in both batches simultaneously (**Supplementary Figure S4**).

Supplementary tables

Supplementary table S1: Normalized main lipid sub-classes distribution in FOLFOXIRI-naïve and FOLFOXIRI-resistant (-R) clones.

		Abundance (%)							
**Main lipid families**	**Ontology**	**DLD1**	**DLD1-R**	**HCT116**	**HCT116-R**	**LS174T**	**LS174T-R**	**SW620**	**SW620-R**
**Triglycerides**	**TG**	10.1	13.4	18.2	30.6	15.3	13.2	37.9	64.9
**Sphingolipids**	**Cer**	4.2	5.1	2.3	2.1	5.3	8.6	1.6	0.5
	**SM**	9.3	8.5	4.9	4.7	3.0	3.5	1.3	0.5
**Phospholipids**	**PC**	52.4	42.8	49.7	44.5	52.1	47.5	40.8	28.7
	**PE**	20.5	25.9	22.2	16.7	18.4	20.5	14.8	4.3
	**PG**	0.0	0.0	0.2	0.0	0.0	0.0	0.0	0.0
	**PI**	0.6	1.0	0.4	0.2	0.6	0.7	0.7	0.2
	**PS**	1.4	2.4	0.7	0.6	2.3	2.6	1.6	0.3
**Other**	**DG**	1.2	0.7	1.1	0.4	2.7	3.1	1.2	0.3
	**other**	0.1	0.1	0.3	0.1	0.2	0.3	0.2	0.2

Supplementary table S2: *Relative variability and block contributions of the AMOPLS model of the data acquired from the investigated biological samples.*

**Effect**	**Relative sum of squares**	**RSR p-value**	**RSR**	**Block contributions (%)**						
				tp1	tp2	tp3	tp4	tp5	tp6	tp7
**Cell origin**	58%	0.01	6.15	**99.6**	**96.4**	0.1	**94.7**	1.0	0.2	3.5
**FOLFOXIRI-Resistance**	7.8%	0.01	1.81	0.1	1.0	**99.3**	1.5	3.3	0.7	11.8
**Origin x Resistance**	18.1%	0.01	2.37	0.1	0.8	0.2	1.1	**89.8**	**97.7**	**63.4**
**Residuals**	15.7%	NA	1	0.2	1.8	0.5	2.7	6.0	1.3	21.4
**Explained variance**				**39.7**	**14.9**	**7.8**	**3.8**	**12.3**	**5.8**	**0.1**

RSR: residual structure ratio, tp: predictive components. The highest contribution is reported in bold fond.

Supplementary table S3: *Analytical sequence with the consecutive SW/LS and HCT/DLD batches with respective QC, conditioning QCs (condQCs) and diluted QCs (dilQCs - dilution by a factor 2).*

**Sample name**	**Batch**	**Group/Sample type**	**Replicate**	**Injection number**
condA.QC11	SW/LS	condQC	NA	016
condA.QC12	SW/LS	condQC	NA	017
condA.QC13	SW/LS	condQC	NA	018
condA.QC14	SW/LS	condQC	NA	019
condA.QC15	SW/LS	condQC	NA	020
QCSWLS01	SW/LS	QC	NA	021
dilQCSWLS01	SW/LS	dilQC	NA	022
QCSWLS02	SW/LS	QC	NA	023
LS.2	SW/LS	LS	2	024
LSR.3	SW/LS	LSR	3	025
SW.1	SW/LS	SW	1	026
SWR.3	SW/LS	SWR	3	027
QCSWLS03	SW/LS	NA	NA	028
LS.1	SW/LS	LS	1	029
LSR.2	SW/LS	LSR	2	030
SW.3	SW/LS	SW	3	031
SWR.1	SW/LS	SWR	1	032
LS.3	SW/LS	LS	3	033
LSR.1	SW/LS	LSR	1	034
SW.2	SW/LS	SW	2	035
SWR.2	SW/LS	SWR	2	036
QCSWLS05	SW/LS	QC	NA	037
dilQCSWLS02	SW/LS	dilQC	NA	038
QCSWLS06	SW/LS	QC	NA	039
condB.QC16	HCT/DLD	condQC	NA	062
condB.QC17	HCT/DLD	condQC	NA	063
condB.QC18	HCT/DLD	condQC	NA	064
condB.QC19	HCT/DLD	condQC	NA	065
condB.QC20	HCT/DLD	condQC	NA	066
QCHCDL01	HCT/DLD	QC	NA	067
dilQCHCDL01	HCT/DLD	dilQC	NA	068
QCHCDL02	HCT/DLD	QC	NA	069
HCT.2	HCT/DLD	HCT	2	070
HCTR.1	HCT/DLD	HCTR	1	071
DLD.1	HCT/DLD	DLD	1	072
DLDR.3	HCT/DLD	DLDR	3	073
QCHCDL03	HCT/DLD	NA	NA	074
HCT.1	HCT/DLD	HCT	1	075
HCTR.3	HCT/DLD	HCTR	3	076
DLD.2	HCT/DLD	DLD	2	077
DLDR.1	HCT/DLD	DLDR	1	078
HCT.3	HCT/DLD	HCT	3	079
HCTR.2	HCT/DLD	HCTR	2	080
DLD.3	HCT/DLD	DLD	3	081
DLDR.2	HCT/DLD	DLDR	2	082
QCHCDL04	HCT/DLD	QC	NA	083

dilQCHCDL02	HCT/DLD	dilQC	NA	084
QCHCDL05	HCT/DLD	QC	NA	085
blank	Blank	Blank	NA	088

Supplementary figures

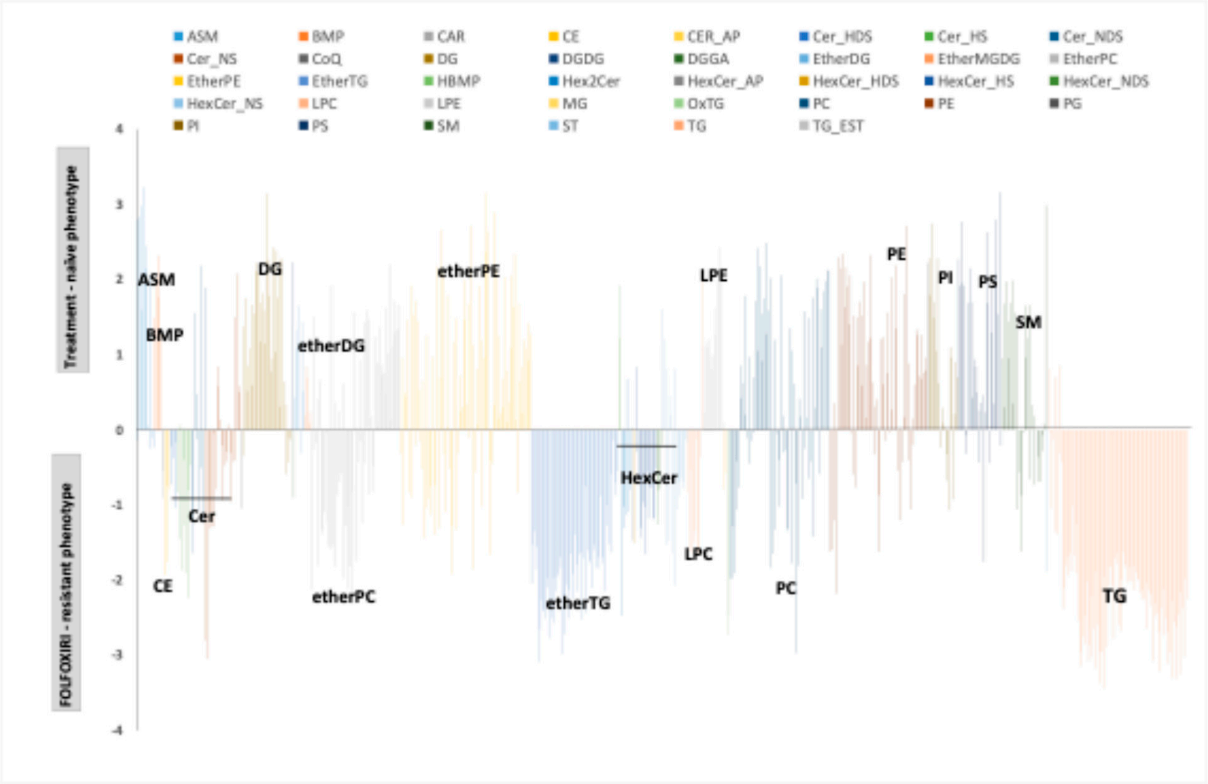

Supplementary figure S1. *Loading plot of AMOPLS predictive component linked to the common resistance main effect (pp3) between all cell lines.*

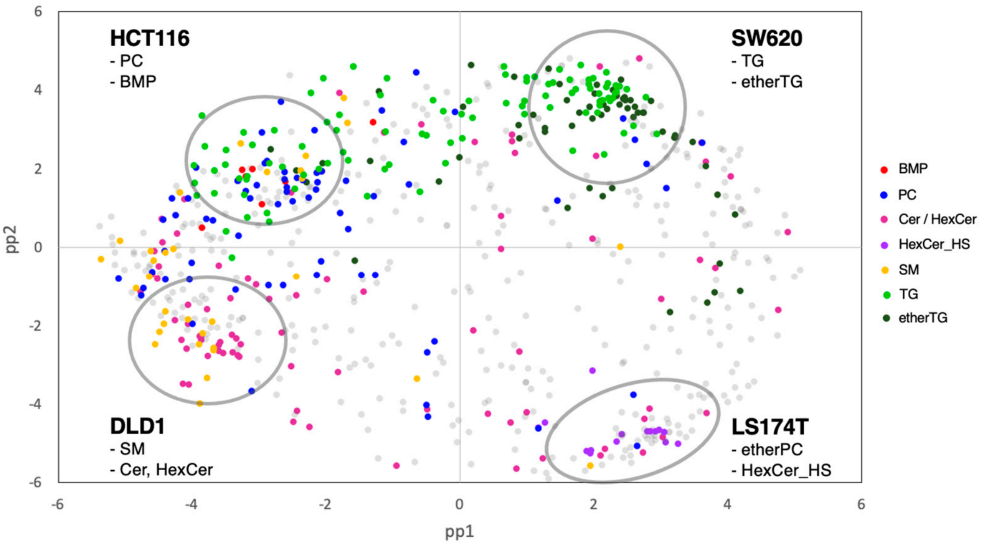

Supplementary figure S2. *AMOPLS predictive components linked to the cell origin main effect.*

Loading plot of cell origin factors pp1 vs. pp2 with lipids most relevant to each of the lines colored by class.

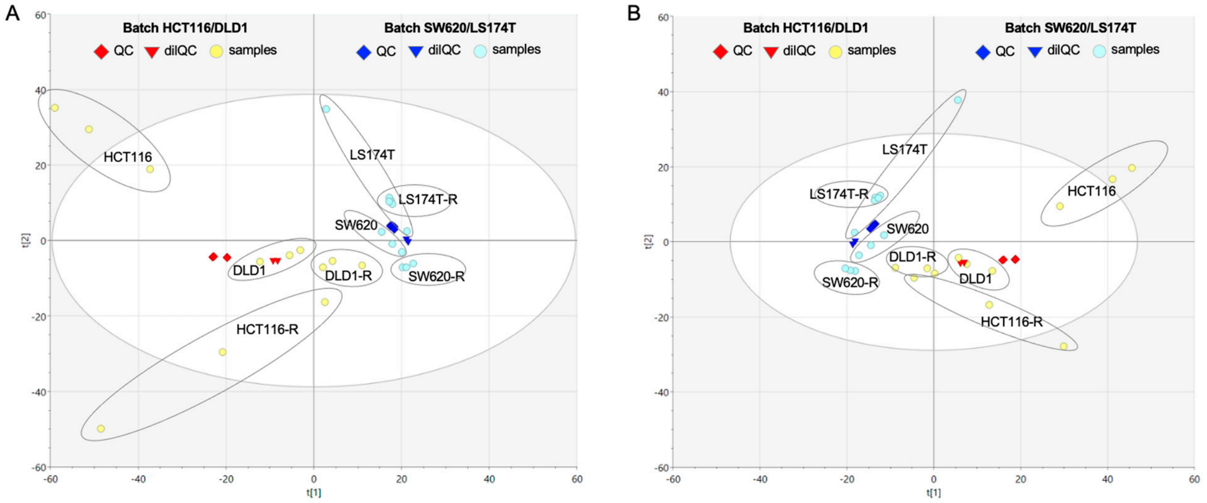

Supplementary figure S3. *PCA score plots of raw* ***(A)*** *and filtered* ***(B)*** *datasets prior PQN correction representing the 2-batch analytical sequence: batch SW620/LS174T with samples in yellow dots, QCs in red lozenges and diluted QCs in red triangles; batch HCT116/DLD1 with samples in light blue, QCs in blue lozenges and diluted QCs in blue triangles.*

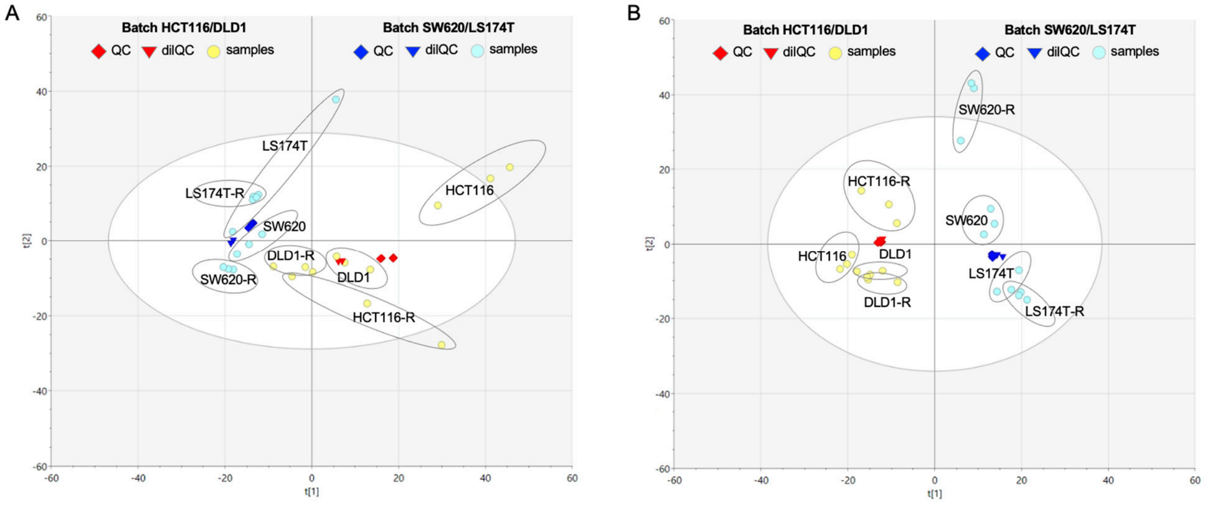

Supplementary figure S4. *PCA score plots of filtered datasets prior* ***(A)*** *vs. after* ***(B)*** *PQN correction.*
